# Association of Nasopharynx Cancer with Human Papillomavirus Infections

**DOI:** 10.3390/cancers15164082

**Published:** 2023-08-13

**Authors:** Shih-Han Hung, Tzong-Hann Yang, Yen-Fu Cheng, Chin-Shyan Chen, Herng-Ching Lin

**Affiliations:** 1 Department of Otolaryngology, School of Medicine, Taipei Medical University, Taipei 110, Taiwan; seedturtle@gmail.com; 2 Department of Otolaryngology, Wan Fang Hospital, Taipei 110, Taiwan; 3 International Ph.D. Program in Medicine, College of Medicine, Taipei Medical University, Taipei 110, Taiwan; 4 Department of Otorhinolaryngology, Taipei City Hospital, Taipei 110, Taiwan; tzonghannyang@gmail.com; 5 Department of Speech, Language and Audiology, National Taipei University of Nursing and Health, Taipei 112, Taiwan; 6 Department of Otolaryngology-Head and Neck Surgery, School of Medicine, National Yang-Ming University, Taipei 112, Taiwan; entist@gmail.com; 7 Center of General Education, University of Taipei, Taipei 112, Taiwan; 8 Research Center of Sleep Medicine, College of Medicine, Taipei Medical University, Taipei 110, Taiwan; stan@mail.ntpu.edu.tw; 9 Department of Medical Research, Taipei Veterans General Hospital, Taipei 112, Taiwan; 10 Department of Otolaryngology-Head and Neck Surgery, Taipei Veterans General Hospital, Taipei 112, Taiwan; 11 Department of Economics, National Taipei University, New Taipei City 237, Taiwan; 12 School of Health Care Administration, College of Management, Taipei Medical University, Taipei 110, Taiwan; 13 Research Center of Sleep Medicine, Taipei Medical University Hospital, Taipei 110, Taiwan

**Keywords:** nasopharyngeal carcinoma, epidemiology, human papillomavirus, human papillomavirus infection

## Abstract

**Simple Summary:**

This study aims to examine the association between nasopharyngeal carcinoma and human papillomavirus infections by means of a nationwide population-based study. This study included 2747 individuals aged 20 years and older who were diagnosed with nasopharynx cancer as cases and 13,735 propensity-score-matching controls. The chi-squared test indicated a significant dissimilarity in previous human papillomavirus infection rates between nasopharynx cancer patients and controls (12.7% vs. 7.2%, *p* < 0.001). The adjusted odds ratio for prior human papillomavirus infections was found to be significantly higher for nasopharyngeal carcinoma cases compared to controls at a value of 1.869 with confidence interval ranging from 1.640 to 2.128 (*p* < 0.001). Our study indicates a noteworthy association between previous human papillomavirus infections and nasopharyngeal carcinoma. Healthcare providers should consider patients’ history of human papillomavirus infection when evaluating their susceptibility to nasopharyngeal carcinoma.

**Abstract:**

This population-based study aims to examine the association between nasopharyngeal carcinoma and human papillomavirus infections. This study included 2747 individuals aged 20 years and older who were diagnosed with nasopharynx cancer as cases and 13,735 propensity-score-matching controls. Multivariate logistic regression models were employed to quantitatively assess the association of nasopharynx cancer with human papillomavirus infections while considering age, sex, monthly income, geographic location, and urbanization level of the patient’s residence as well as diabetes, hypertension, and hyperlipidemia. Our chi-squared test indicated a significant dissimilarity in previous human papillomavirus infection rates between nasopharynx cancer patients and controls (12.7% vs. 7.2%, *p* < 0.001). The adjusted odds ratio (OR) for prior human papillomavirus infections was found to be significantly higher for nasopharyngeal carcinoma cases compared to controls at a value of 1.869 with confidence interval ranging from 1.640 to 2.128. Among female participants, compared to controls, the adjusted OR of prior human papillomavirus infections was 2.150 (95% CI = 1.763–2.626) in patients with nasopharynx cancer. In male participants sampled in this study, we observed a statistically significant association between prior human papillomavirus infections and nasopharynx cancer (adjusted OR = 1.689; 95% CI = 1.421–2.008). Our study indicates a noteworthy association between previous human papillomavirus infections and nasopharyngeal carcinoma.

## 1. Introduction

Nasopharyngeal carcinoma is characterized by a multifaceted etiology, with its incidence being particularly elevated in specific populations such as indigenous individuals of southern China, Southeast Asia, the Arctic, and the Middle East/North Africa. This unique racial/ethnic and geographic distribution suggests that both environmental factors and genetic traits are involved in its development [1]. In areas where nasopharyngeal carcinoma is prevalent, undifferentiated subtypes constitute most cases and are consistently linked to Epstein–Barr virus (EBV) infection [2]. EBV-related genetic and epigenetic mechanisms play a crucial role in nasopharyngeal carcinoma pathogenesis [3,4,5].

Similarly, the human papillomavirus, also known as highly oncogenic, is associated with numerous cancer types, such as those of the cervix, anus, genitals, and throat [6,7]. High-risk HPVs are believed to be linked to approximately 3% of cancers in women and 2% in men. The causal correlation between human papillomavirus infections and cervical cancer has been firmly established since this association exists worldwide in virtually all cervical cancer cases [8]. According to reports, an estimated total of 110,650 new cancer cases and 36,714 deaths attributable to human papillomavirus infection occurred in China in 2015 [9].

High-risk human papillomavirus is now recognized as the primary offender behind the escalating incidence rates of oropharyngeal squamous cell carcinoma (OPSCC) in certain regions around the world [10]. Despite decades of research on EBV viral-oncogenesis concerning nasopharyngeal carcinoma, little attention has been paid to exploring the potential involvement of human papillomavirus in the development of nasopharyngeal carcinoma. One earlier study discovered that a positive history of cervical cancer among first-degree relatives was linked with an increased risk for nasopharyngeal carcinoma [1]. Numerous clinical studies have further isolated human papillomavirus from nasopharyngeal carcinoma tumor tissues, with the highest prevalence reported up to 31% [11,12,13,14]. This prompts us to investigate the existence of a significant association between HPV and nasopharyngeal carcinoma. This study aims to examine the association between nasopharyngeal carcinoma and human papillomavirus infections by means of a nationwide population-based study, thereby enhancing comprehension of nasopharyngeal carcinoma etiology and potentially enlightening preventive and clinical approaches.

## 2. Methods

### 2.1. Database

We extracted data from Taiwan’s Longitudinal Health Insurance Database 2010 for this retrospective observational study. In 1995, Taiwan introduced a single-payer compulsory social healthcare insurance program, the National Health Insurance (NHI) program. This program offers comprehensive medical care with low copayments to all Taiwanese citizens. The medical claim data under the NHI program are released as the National Health Insurance Research Database. The National Health Insurance Research Database consists of a de-identified registry for beneficiaries, ambulatory care claims, inpatient claims, prescriptions dispensed at pharmacies, registry for medical facilities, and registry for board-certified specialists primarily used for reimbursement purposes. The Data Science Centre of the Ministry of Health and Welfare of Taiwan has created a number of sub-datasets within the National Health Insurance Research Database, including the Longitudinal Health Insurance Database 2010. The Longitudinal Health Insurance Database 2010 comprises beneficiary registration files and medical claims files of a random sample of two million NHI beneficiaries. Numerous researchers and scholars have utilized the Longitudinal Health Insurance Database 2010 for epidemiological studies on diseases and their treatments.

The study was approved by the institutional review board of Taipei Medical University (TMU-JIRB N202208042) and is compliant with the Declaration of Helsinki. The Data Science Centre of the Ministry of Health and Welfare of Taiwan has encrypted the names of patients, healthcare providers, and medical institutions with unique and anonymous identifiers to protect patients’ privacy. Therefore, the Longitudinal Health Insurance Database 2010 is a de-identified administrative dataset, so patient informed-consent was waived in this study.

### 2.2. Identification of Study Patients

This study was designed as a case-control study. For the purpose of selecting cases in this case-control study, we identified 2747 individuals aged 20 years and older who were diagnosed with nasopharyngeal carcinoma (ICD-9-CM 147, ICD-10-CM C11) for the first time between 1 January 2012 and 31 December 2019. Furthermore, we determined the index date as the day on which they received their initial diagnosis of nasopharyngeal carcinoma.

To investigate the association between human papillomavirus infections and nasopharyngeal carcinoma, we used a propensity score matching technique to identify appropriate controls from the remaining beneficiaries aged 20 years and above in the Longitudinal Health Insurance Database 2010 registry. Any individuals with a history of nasopharynx cancer in their medical records were excluded from our analysis. Propensity scores were computed for all 2747 patients with nasopharynx cancer and the remaining beneficiaries using logistic regression models that matched for the variables of sociodemographic characteristics and the most prevalent medical co-morbidities in Taiwan, including age, gender, monthly income (TWD 0–15,840, TWD 15,841–25,000, ≥TWD 25,001; USD 1 ≈ TWD 28 in 2021), geographic location (Northern, Central, Southern, and Eastern), urbanization level of patient’s residence (5 levels where level 1 is most urbanized and level 5 least urbanized), diabetes, hypertension, and hyperlipidemia, and the year of the index date. For cases, we designated the year of the index date as the year they were first diagnosed with nasopharynx cancer. Conversely, the index date for controls was simply a matched year in which they received ambulatory care.

Finally, each sampled patient with nasopharyngeal carcinoma was matched for five controls without nasopharyngeal carcinoma through the nearest neighbor random matching algorithm with caliper adjustment, using a priori value for the calipers of +/− 0.01. Consequently, our study comprised 2747 patients with nasopharyngeal carcinoma and 13,735 control subjects without this condition.

### 2.3. Measures of Outcomes

We identified cases of human papillomavirus infections through the use of ICD-9-CM codes 078.10, 078.11, 078.12, 078.19, 795.1, and 079.4, along with records indicating positive results from polymerase chain reaction tests. ICD-10-CM codes B07.08, B97.7, R85.81, R85.82, R87.82, and A63 were considered as well for inclusion in this study’s analysis process only if they had received at least one diagnosis of HPV infection prior to the index date under consideration here.

The study utilized a combination of clinical diagnosis codes and molecular diagnosis codes, including the PCR test, to identify cases of HPV infection. This approach was chosen because the PCR test is not mandatory for managing related diseases in our healthcare system. In order to include as many cases of HPV as possible and further explore the potential relationship with NPC, the study design may result in heterogeneity in the diagnosis of HPV. As only a portion of patients underwent HPC PCR testing, we were unable to analyze specific types of HPV within our study.

### 2.4. Statistical Analysis

The statistical analyses were conducted using the SAS system (SAS System for Windows, ver. 9.4, SAS Institute, Cary, NC, USA). To investigate differences in baseline characteristics between patients with nasopharyngeal carcinoma and controls, we utilized chi-square tests and Mann–Whitney U tests. Additionally, multivariate logistic regression models were employed to quantitatively assess the association of nasopharyngeal carcinoma with human papillomavirus infections while considering age, sex, monthly income, geographic location, and urbanization level of the patient’s residence as well as diabetes, hypertension, and hyperlipidemia. The estimated odds ratios along with their corresponding 95% confidence intervals were used to determine disparities in the odds of human papillomavirus infections among patients who have nasopharyngeal carcinoma relative to those without it. All *p*-values were two-sided; a *p*-value < 0.05 was considered statistically significant.

## 3. Results

The mean age of the study population was 56.4 years, with a standard deviation of 14.5 years. Table 1 indicates that, after employing propensity score matching techniques, there were no significant differences between patients afflicted with nasopharyngeal carcinoma and controls in terms of age (*p* = 0.959), gender (*p* > 0.999), level of urbanization (*p* = 0.994), monthly income (*p* = 0.909), and geographic region (*p* = 0.602). Additionally, there were no statistically significant disparities detected in the prevalence of diabetes (*p* > 0.999), hypertension (*p* > 0.999), or hyperlipidemia (*p* > 0.999) among patients who have nasopharyngeal carcinoma compared to their control counterparts.

Table 2 depicts the frequency of prior human papillomavirus infections among the sampled patients. Our analysis revealed that out of 16,482 individuals assessed, 1342 (8.1%) had previously contracted human papillomavirus before the index date. Our chi-squared test indicated a significant dissimilarity in previous HPV infection rates between patients with nasopharyngeal carcinoma and controls (12.7% vs. 7.2%, *p* < 0.001).

Furthermore, Table 2 presents the results of multivariate logistic regression analyses where we discovered that after adjusting for age, sex, monthly income, geographic location, and urbanization level of residence, as well as comorbidities such as diabetes, hypertension, and hyperlipidemia, the odds ratio for prior human papillomavirus infections was found to be significantly higher for patients with nasopharyngeal carcinoma compared to controls, at a value of 1.869 with confidence interval ranging from 1.640 to 2.128 (*p* < 0.001).

Table 3 presents the prevalence and adjusted odds ratios of prior human papillomavirus infections among patients with nasopharyngeal carcinoma compared to controls stratified by sex. Among female participants, after adjusting for age, sex, monthly income, geographic location, urbanization level of residence, diabetes, hypertension, and hyperlipidemia, the odds ratio of prior human papillomavirus infections was 2.150 (95% confidence interval = 1.763–2.626) in patients with nasopharyngeal carcinoma relative to controls. In male participants sampled in this study, we observed a statistically significant association between prior human papillomavirus infections and nasopharyngeal carcinoma (adjusted odds ratio = 1.689; 95% confidence interval = 1.421–2.008). 

## 4. Discussion

This nationwide population-based investigation explored the linkage between nasopharyngeal carcinoma and human papillomavirus infections. Our results indicate a significant correlation between prior human papillomavirus infections and nasopharyngeal carcinoma. After adjusting for age, gender, income per month, geographical location, urbanization level of residency, as well as comorbidities including diabetes mellitus, hypertension, and hyperlipidemia, the OR for prior human papillomavirus infections was found to be substantially higher in cases with nasopharyngeal carcinoma than controls (odds ratio = 1.869; 95% confidence interval = 1.640–2.128; *p* < 0.001). This relationship was observed in both male and female participants, with females exhibiting a stronger association than males (odds ratio = 2.150; 95% confidence interval = 1.763–2.626 vs. odds ratio = 1.689; 95% confidence interval = 1.421–2.008, respectively).

Comprehending the correlation between nasopharyngeal carcinoma and human papillomavirus infections holds immense significance. It imparts valuable knowledge about the origin of nasopharyngeal carcinoma, which could pave the way for prophylactic measures and precise therapies. Additionally, it could aid in detecting high-risk individuals who might benefit from human papillomavirus immunization and guide screening approaches, as those with prior human papillomavirus infections may necessitate more frequent monitoring for nasopharyngeal carcinoma. Numerous inquiries have been conducted to explore the correlation between human papillomavirus and head and neck malignancies. Deng et al.’s research revealed that individuals diagnosed with head and neck squamous cell carcinoma (HNSCC) who contracted human papillomavirus experienced a more favorable prognosis than those without human papillomavirus infection [15]. Maruyama et al.’s study indicated that HNSCC could be classified into virus-related and virus-unrelated subcategories based on its etiology [16]. Lo et al. explored the correlation between human papillomavirus and World Health Organization Type I human papillomavirus, proposing a potential association [17].

Several studies have been conducted on the prevalence and pattern of human papillomavirus (HPV) infection in nasopharyngeal carcinomas. One such study was carried out by Dogan et al. in a low-incidence population, where they aimed to investigate the occurrence and characteristics of HPV infection [18]. The authors concluded that the etiologic role of HPV in NPC is confirmed, and the favorable prognostic significance of HPV positivity is similar to that of EBV positivity. Another study performed by Tham et al. was a systematic review and meta-analysis that explored the prognostic implications of HPV status for nasopharyngeal carcinoma [19]. The researchers further reported significant evidence to support this relationship, prompting them to examine the distribution of Epstein–Barr virus (EBV) and HPV based on the histological type of nasopharyngeal carcinoma. Their findings revealed that HPV DNA had a higher prevalence in WHO Type I (34.4%) compared to WHO Type II/III (18.4%) [20]. These collective results provide valuable insights into understanding the relationship between HPV and the pathogenesis of nasopharyngeal carcinoma, which can aid in developing effective prevention and treatment strategies for this disease.

Human papillomavirus has been implicated in the development of nasopharyngeal cancer through various mechanisms. The virus, especially high-risk strains like HPV-16 and HPV-18, encode oncoproteins E6 and E7 that can disrupt standard cellular cycle regulation. The E6 protein binds to and stimulates the degradation of tumor suppressor protein p53, thereby inhibiting its role in DNA repair and apoptosis. Simultaneously, the E7 protein interacts with retinoblastoma (Rb) protein, liberating transcription factor E2F to promote cell proliferation. Collectively, these actions result in genomic instability and hold potential for malignant transformation. Additionally, human papillomavirus is known to trigger chronic inflammation in the nasopharyngeal epithelium, which could contribute to carcinogenesis. It is noteworthy that human papillomavirus can integrate into the host genome thus promoting further oncogenic activity [21].

Mostafaei et al. recently revealed a substantial correlation between the EBV and human papillomavirus genes, which jointly contribute to anoikis resistance and the onset of breast and thyroid cancers. This was demonstrated through simultaneous SEM analysis [22]. Nevertheless, it is crucial to investigate whether the observed increase in OR in this study exclusively arises from human papillomavirus or if a synergistic effect exists with EBV. It should be emphasized that co-infection of human papillomavirus and EBV in nasopharyngeal carcinomas appears rare [23].

Notably, our investigation revealed that the female population exhibited a significantly higher odds ratio for previous human papillomavirus infections in nasopharyngeal carcinoma cases than controls. Specific inquiries have suggested that females may be more susceptible to human papillomavirus-induced cancers due to limited awareness and knowledge of the virus and its potential effects [24]. Arbeit et al., utilizing a mouse model, demonstrated a unique mechanism of synergistic collaboration between chronic estrogen exposure and HPV16 oncogenes which coordinates squamous carcinogenesis in the female reproductive tract [25]. Recent research has indicated that estrogen signaling plays a crucial role in human papillomavirus-positive cancers [26]. These findings offer valuable insight into elucidating gender disparities observed within our study.

The discoveries that we have made carry significant clinical implications that could potentially impact the way we approach certain health risks. Firstly, our findings suggest that human papillomavirus infection may act as a risk factor for nasopharyngeal carcinoma, which is an important piece of information that could inform screening and risk assessment strategies for this type of cancer. This is particularly relevant given the fact that nasopharyngeal carcinoma is not commonly screened for in routine check-ups, meaning that many cases go undetected until they have reached an advanced stage. Secondly, our research suggests that these findings could potentially facilitate the development of preventive measures such as human papillomavirus vaccination for individuals at a higher risk of developing nasopharyngeal carcinoma. This is especially important because no specific vaccines are currently available for this type of cancer. In their hypothesis paper, Chan et al. noted that regional disease burdens of oncogenic strains of human papillomavirus are strongly associated with type-specific risks of nasopharyngeal carcinoma based on data from the literature [27]. If these concerns prove to be true, it will open up potential preventive interventions such as community human papillomavirus control and newborn vaccination as viable options. This would be a major breakthrough in the fight against nasopharyngeal carcinoma and could help significantly reduce this disease’s incidence rate.

Overall, our discoveries have opened up new possibilities for preventing and managing nasopharyngeal carcinoma by shedding light on the role played by human papillomavirus infection in its development. By exploring these connections further and developing effective prevention strategies, we can hope to reduce the burden of this disease on individuals and healthcare systems alike. Also noteworthy are the possible influences of the parallel burden of oncogenic HPV and EBV infections in developing nasopharynx cancers. It is yet to be determined whether the HPV and EBV viruses have a synergistic effect or compete against each other in driving NPC development. Dogan’s study has shown that HPV positivity yields a similar favorable prognosis as EBV, thus prompting further investigation into the potential prognostic impact of co-infection with both viruses.

Finally, these findings could facilitate the advancement of tailored treatments for nasopharyngeal carcinoma. Recently disclosed molecular pathways have shed light on targeted therapeutic approaches targeting E proteins and suppressing human papillomavirus oncoproteins [28]. Additional research is warranted to ascertain the potential of implementing human papillomavirus-targeted therapies in individuals with human papillomavirus-positive nasopharyngeal carcinoma.

Despite its strengths, our study has several limitations to consider when interpreting the results. Firstly, the study design is retrospective and observational, which inherently limits our ability to infer causality from the observed associations. Secondly, we relied on administrative data from the Longitudinal Health Insurance Database 2010, which may be subject to coding errors and misclassifications. Thirdly, we did not have information on specific potential confounders, such as lifestyle factors (e.g., smoking and alcohol consumption), dietary habits, and family history of cancer, which could influence both the risk of human papillomavirus infection and nasopharyngeal carcinoma. Fourthly, our study was conducted in Taiwan, and the findings may not be generalizable to other populations with different genetic backgrounds and environmental exposures. Lastly, the detection of human papillomavirus infections was based on medical records, which might be influenced by the frequency of medical visits and testing practices, potentially leading to detection bias. Despite these limitations, our study provides valuable insight into the association between human papillomavirus infections and nasopharyngeal carcinoma, which warrants further investigation in future studies.

In order to build upon and confirm our research findings, it is essential for future studies to explore the relationship between human papillomavirus (HPV) infections and nasopharyngeal carcinoma in diverse populations and settings. To achieve this, researchers should consider investigating the potential mechanisms underlying this connection through molecular and genetic studies that can help clarify how HPV infection contributes to the development of nasopharyngeal carcinoma. Moreover, it would be worthwhile to examine the potential benefits of HPV vaccination in reducing the risk of nasopharyngeal carcinoma. This could involve conducting randomized controlled trials to assess the efficacy of HPV vaccines in preventing nasopharyngeal carcinoma, especially in groups with a high prevalence of HPV infections. Additionally, developing predictive models that incorporate HPV infection status along with other risk factors could help enhance early detection and prevention of nasopharyngeal carcinoma. Lastly, health economic evaluations should be conducted to assess the cost-effectiveness of different preventive strategies, such as HPV vaccination and screening programs, in reducing the burden of nasopharyngeal carcinoma. By taking these steps forward in research, we can advance our understanding of how best to prevent and manage this disease for individuals across diverse populations and settings.

After conducting our research, we strongly recommend that clinicians take into account a patient’s history of human papillomavirus infections when assessing their risk for nasopharyngeal carcinoma. Our findings have shown a clear association between human papillomavirus infections and nasopharyngeal carcinoma, indicating that individuals who have had previous infections may require more frequent monitoring to prevent the development of this type of cancer. Additionally, we believe that human papillomavirus vaccination could be an effective prevention strategy for nasopharyngeal carcinoma, especially in high-risk patients. However, further studies are necessary to confirm this potential benefit. In light of these results, healthcare providers must educate their patients about the potential risks associated with human papillomavirus infections, including the increased risk of developing nasopharyngeal carcinoma. Patients should also be encouraged to practice safe sexual habits and consider receiving the human papillomavirus vaccine as a preventive measure. By taking these proactive steps, we can work towards reducing the incidence and impact of nasopharyngeal carcinoma caused by human papillomavirus infections.

## 5. Conclusions

In conclusion, our study indicates a noteworthy association between previous human papillomavirus infections and nasopharyngeal carcinoma. These results emphasize the potential contribution of human papillomavirus in the pathogenesis of nasopharyngeal carcinoma and accentuate the necessity for additional investigations in this domain. Healthcare providers should consider patients’ history of human papillomavirus infection when evaluating their susceptibility to nasopharyngeal carcinoma.

## Figures and Tables

**Table 1:** Demographic characteristics of patients with nasopharynx cancer and propensity score-matched controls (*n* = 16,482).

Variable	Patients with Nasopharynx Cancer (*n* = 2747)		Propensity Score-Matched Controls (*n* = 13,735)		*p* Value
	Total No.	%	Total No.	%	
Age, mean (SD)	56.4 (14.5)		56.4 (14.5)		0.959
Males	1749	63.7	8745	63.7	>0.999
Monthly income					0.909
<TWD 1–15,841	561	20.4	2786	20.3	
TWD 15,841–25,000	907	33.0	4594	33.4	
≥TWD 25,001	1279	46.6	6355	46.3	
Geographic region					0.602
Northern	1258	45.8	6312	46.0	
Central	706	25.7	3488	25.4	
Southern	728	26.5	3707	27.0	
Eastern	55	2.0	228	1.6	
Urbanization level					0.994
1 (most urbanized)	757	27.6	3819	27.8	
2	803	29.2	4021	29.3	
3	489	17.8	2423	17.6	
4	369	13.4	1862	13.6	
5 (least urbanized)	329	12.0	1610	11.7	
Hypertension	1105	40.2	5525	40.2	>0.999
Hyperlipidemia	986	35.9	4930	35.9	>0.999
Diabetes	657	23.9	3285	23.9	>0.999

**Table 2:** Prevalence rates and odds ratio of HPV among patients with nasopharynx cancer vs. controls.

Presence of HPV	Total (*n* = 16,482)		Patients with Nasopharynx Cancer (*n* = 2747)		Controls (*n* = 13,735)	
			*n*, %			
Yes	1342	8.1	348	12.7	994	7.2
No	15,140	91.9	2399	87.3	12,741	92.8
Odds ratio ^a^ (95% CI)	-		1.869 * (1.640–2.128)		1.000	

Notes: ^a^ Adjusted for age, monthly income, geographic location, urbanization level, hyperlipidemia, diabetes, and hypertension; * *p* < 0.001.

**Table 3:** Prevalence rates and odds ratio of HPV among patients with nasopharynx cancer vs. controls according to sex.

**Males**						
**Presence of HPV**	**Total (*n* = 10,494)**		**Patients with Nasopharynx Cancer (*n* = 1749)**		**Controls (*n* = 8745)**	
	***n*, %**					
Yes	781	7.4	190	10.9	591	6.8
No	9713	92.6	1559	89.1	8154	93.2
Odds ratio ^a^ (95% CI)	-		1.689 * (1.421–2.008)		1.000	
**Females**						
**Presence of HPV**	**Total (*n* = 5988)**		**Patients with Nasopharynx Cancer (*n* = 998)**		**Controls (*n* = 4990)**	
	***n*, %**					
Yes	561	9.4	158	15.8	403	8.0
No	5427	90.6	840	84.2	4587	92.0
Odds ratio ^a^ (95% CI)			2.150 * (1.763–2.622)		1.000	

Notes: ^a^ Adjusted for age, monthly income, geographic location, urbanization level, hyperlipidemia, diabetes, and hypertension; * *p* < 0.001.

## Data Availability

Data from the National Health Insurance Research Database, now managed by the Health and Welfare Data Science Center (HWDC), can be obtained by interested researchers through a formal application process addressed to the HWDC, Department of Statistics, Ministry of Health and Welfare, Taiwan (https://dep.mohw.gov.tw/DOS/lp-2506-113.html, accessed on 2 January 2022).

## References

[1] Chang E.T., Adami H.-O. (2006). The enigmatic epidemiology of nasopharyngeal carcinoma. Cancer Epidemiol. Biomark. Prev..

[2] Nakanishi Y., Wakisaka N., Kondo S., Endo K., Sugimoto H., Hatano M., Ueno T., Ishikawa K., Yoshizaki T. (2017). Progression of understanding for the role of Epstein-Barr virus and management of nasopharyngeal carcinoma. Cancer Metastasis Rev..

[3] Zhang Y., Fan J., Fan Y., Li L., He X., Xiang Q., Mu J., Zhou D., Sun X., Yang Y. (2018). The new 6q27 tumor suppressor DACT2, frequently silenced by CpG methylation, sensitizes nasopharyngeal cancer cells to paclitaxel and 5-FU toxicity via β-catenin/Cdc25c signaling and G2/M arrest. Clin. Epigenetics.

[4] Raab-Traub N. (2002). Epstein–Barr Virus in the Pathogenesis of NPC. Seminars in Cancer Biology.

[5] Dawson C.W., Port R.J., Young L.S. (2012). The role of the EBV-encoded latent membrane proteins LMP1 and LMP2 in the pathogenesis of nasopharyngeal carcinoma (NPC). Seminars in Cancer Biology.

[6] Senkomago V., Henley S.J., Thomas C.C., Mix J.M., Markowitz L.E., Saraiya M. (2019). Human papillomavirus–attributable cancers—United States, 2012–2016. Morb. Mortal. Wkly. Rep..

[7] Economopoulou P., Kotsantis I., Psyrri A. (2020). Special issue about head and neck cancers: HPV positive cancers. Int. J. Mol. Sci..

[8] Bosch F.X., Lorincz A., Muñoz N., Meijer C.J., Shah K.V. (2002). The causal relation between human papillomavirus and cervical cancer. J. Clin. Pathol..

[9] Lu Y., Li P., Luo G., Liu D., Zou H. (2020). Cancer attributable to human papillomavirus infection in China: Burden and trends. Cancer.

[10] Taberna M., Mena M., Pavón M.A., Alemany L., Gillison M.L., Mesía R. (2017). Human papillomavirus-related oropharyngeal cancer. Ann. Oncol..

[11] Isayeva T., Li Y., Maswahu D., Brandwein-Gensler M. (2012). Human papillomavirus in non-oropharyngeal head and neck cancers: A systematic literature review. Head Neck Pathol..

[12] Lo E.J., Bell D., Woo J.S., Li G., Hanna E.Y., El-Naggar A.K., Sturgis E.M. (2010). Human papillomavirus and WHO type I nasopharyngeal carcinoma. Laryngoscope.

[13] Laantri N., Attaleb M., Kandil M., Naji F., Mouttaki T., Dardari R., Belghmi K., Benchakroun N., El Mzibri M., Khyatti M. (2011). Human papillomavirus detection in moroccan patients with nasopharyngeal carcinoma. Infect. Agents Cancer.

[14] Hørding U., Nielsen H.W., Daugaard S., Albeck H. (1994). Human papillomavirus types 11 and 16 detected in nasopharyngeal carcinomas by the polymerase chain reaction. Laryngoscope.

[15] Deng Z., Uehara T., Maeda H., Hasegawa M., Matayoshi S., Kiyuna A., Agena S., Pan X., Zhang C., Yamashita Y. (2014). Epstein-Barr virus and human papillomavirus infections and genotype distribution in head and neck cancers. PLoS ONE.

[16] Maruyama H., Yasui T., Ishikawa-Fujiwara T., Morii E., Yamamoto Y., Yoshii T., Takenaka Y., Nakahara S., Todo T., Hongyo T. (2014). Human papillomavirus and p53 mutations in head and neck squamous cell carcinoma among Japanese population. Cancer Sci..

[17] Lo K.W., Chung GTYTo K.F. (2012). Deciphering the molecular genetic basis of NPC through molecular, cytogenetic, and epigenetic approaches. Seminars in Cancer Biology.

[18] Dogan S., Hedberg M.L., Ferris R.L., Rath T.J., Assaad A.M., Chiosea S.I. (2014). Human papillomavirus and Epstein–Barr virus in nasopharyngeal carcinoma in a low-incidence population. Head Neck.

[19] Tham T., Teegala S., Bardash Y., Herman S.W., Costantino P. (2018). Is human papillomavirus and p16 expression associated with survival outcomes in nasopharyngeal cancer? A systematic review and meta-analysis. Am. J. Otolaryngol..

[20] Tham T., Machado R., Russo D.P., Herman S.W., Teegala S., Costantino P. (2021). Viral markers in nasopharyngeal carcinoma: A systematic review and meta-analysis on the detection of p16INK4a, human papillomavirus (HPV), and Ebstein-Barr virus (EBV). Am. J. Otolaryngol..

[21] Marur S., D’Souza G., Westra W.H., Forastiere A.A. (2010). HPV-associated head and neck cancer: A virus-related cancer epidemic. Lancet Oncol..

[22] Mostafaei S., Kazemnejad A., Norooznezhad A.H., Mahaki B., Moghoofei M. (2020). Simultaneous effects of viral factors of human papilloma virus and Epstein-Barr virus on progression of breast and thyroid cancers: Application of structural equation modeling. Asian Pac. J. Cancer Prev..

[23] Singhi A.D., Califano J., Westra W.H. (2012). High-risk human papillomavirus in nasopharyngeal carcinoma. Head Neck.

[24] Kanmodi K. (2020). Knowledge of HPV, HPV-induced cancers, and HPV vaccine among university students in medical laboratory science disciplines: Nigerian study. J. Obstet. Gynecol. Investig..

[25] Arbeit J.M., Howley P.M., Hanahan D. (1996). Chronic estrogen-induced cervical and vaginal squamous carcinogenesis in human papillomavirus type 16 transgenic mice. Proc. Natl. Acad. Sci. USA.

[26] James C.D., Morgan I.M., Bristol M.L. (2020). The relationship between estrogen-related signaling and human papillomavirus positive cancers. Pathogens.

[27] Chan Y.H., Lo C.M., Lau H.Y., Lam T.H. (2014). Vertically transmitted nasopharyngeal infection of the human papillomavirus: Does it play an aetiological role in nasopharyngeal cancer?. Oral Oncol..

[28] Bhattacharjee R., Das S.S., Biswal S.S., Nath A., Das D., Basu A., Malik S., Kumar L., Kar S., Singh S.K. (2022). Mechanistic role of HPV-associated early proteins in cervical cancer: Molecular pathways and targeted therapeutic strategies. Crit. Rev. Oncol./Hematol..

